# U-shaped prognostic signature: baseline platelet count and morphological parameters predict ovarian cancer outcomes in a 265-patient cohort

**DOI:** 10.3389/fonc.2026.1765566

**Published:** 2026-02-13

**Authors:** Yao Li, Songning Wang, Qiushi Wang, Zhan Li, Cuiqin Sang, Lei Zhu, Shuzhen Wang, Jinfeng Li

**Affiliations:** Department of Obstetrics and Gynecology, Beijing Chaoyang Hospital Affiliated with Capital Medical University, Beijing, China

**Keywords:** ovarian cancer, platelet count, platelet morphological indicator, prognosis, recurrence

## Abstract

**Purpose:**

This study aims to explore the predictive value of baseline platelet count and its morphological indicators for the prognosis of ovarian cancer patients.

**Method:**

A retrospective cohort study was conducted at a gynecological oncology center in Beijing, involving ovarian cancer patients between 2011 and 2022 and followed up until December 2024. Data were extracted from clinical information system. The primary endpoints were recurrence; the primary indicator was progression-free survival during the follow-up period.

**Result:**

A total of 265 patients was included in this study. During the follow-up period, 110 patients recurred, whereas 155 patients achieved remission. Univariate analysis revealed that baseline platelet count was associated with progression-free survival. The stratified analysis presented a U-shaped curve by smooth curve fitting. Threshold effect analysis indicated that the inflection points of the U-shaped curve occurred at platelet count of 236×10^9^/L (95% CI 222-256×10^9^/L), the lowest risk of recurrence. The U-shaped curve was confirmed by Multinomial logistic regression (*P* < 0.005). The relationship between platelet morphological indicators and recurrence risk is modulated by the level of baseline platelet count(*P* < 0.05). In the third tertile of platelet distribution, morphological indicators are associated with recurrence risk and exhibit a protective effect; however, in patients with high-grade serous carcinoma and at different clinical stages, recurrence risk is not significantly associated with morphological indicators; but morphological indicators still have a protective effect on other pathological types (PDW, OR 0.6, *P* = 0.017; MPV, OR 0.3, *P* = 0.025; PLCR, OR 0.9, *P* = 0.022,respectively). The non-parametric Mann-Whitney U test also showed that the predictive value of baseline platelet morphology indicators for recurrence risk was only demonstrated in patients with other pathological types.

**Conclusion:**

This study reveals a significant nonlinear association between platelet count and morphology and the risk of recurrence of ovarian cancer. Exploring the complex mechanisms linking baseline platelet characteristics to the prognosis of ovarian cancer will help facilitate the application of platelets as meaningful prognostic indicators and therapeutic targets in clinical practice.

## 1 Introduction

Epithelial ovarian cancer is the leading cause of death from gynecologic malignancies (1). Most cases are diagnosed at advanced stages, resulting in a 5-year survival rate of approximately 50%. The high mortality rate of ovarian cancer is primarily attributed to the challenges in early diagnosis and the development of chemotherapy resistance (2, 3). Despite significant advancements in poly ADP-ribose polymerase (PARP) inhibitors, anti-angiogenic drugs, and immunotherapy, early diagnosis to ovarian cancer has not been effectively resolved.

Recent studies shown that platelets play a crucial role in tumor initiation and progression. Although the underlying mechanisms remain to be fully elucidated. Studies imply that platelets are related to tumor angiogenesis, epithelial-mesenchymal transition, and immune escape by secreting factors such as platelet-derived growth factor transforming growth factor-β, and platelets activated state is closely related to tumor progression, platelet activation serves as an essential prerequisite, which including the increase of platelet count, release of various biological molecules with morphological alterations (4–7). Electron microscopy has revealed that platelets of patients with ovarian tumors have more mitochondria and significantly smaller microtubules compared to platelets from healthy individuals (8). Researchers from epidemiological studies have found that the incidence of cancer in the year following the assessment of thrombocytosis was 6.2% in females, as compared to 2.2% in females with a normal platelet count (9). The research shown combination platelet count with carbohydrate antigen 125 values at diagnosis, which can improve the diagnostic accuracy (10).

Some studies have demonstrated the clinical significance of platelet count, mean platelet volume (MPV), platelet distribution width (PDW), and large platelet ratio (PLCR)-parameters reflecting platelet morphology-suggesting their potential utility as diagnostic and prognostic markers for various types of cancer (11–13). However, previous investigations on platelet indices as diagnostic or prognostic indicators in cancer have yielded inconsistent results, often derived from small sample sizes, with a predominant focus on platelet count or isolated morphological parameters, while largely overlooking the dynamic interrelationships between platelet count and other morphological indices (14–16). Moreover, there remains a lack of multicenter, large-sample studies. To date, the association between prognosis of ovarian cancer and baseline platelet count and morphological indicators did not been reported.

This study aims to integrate multiple dimensions of indicators, including baseline platelet count, mean platelet volume, platelet distribution width and large platelet ratio, to clarify the quantitative relationship between baseline platelet count and its related volume indicators in the prognosis of ovarian cancer. This study is expected to overcome the limitations of current prognostic assessment in ovarian cancer and identify new targets for precision treatment.

## 2 Methods

### 2.1 Study design

This was a retrospective cohort study. Patients were included from Beijing Chaoyang Hospital if they were diagnosed with ovarian cancer between January 1, 2011, and December 31, 2022. Patients were followed up to the endpoint outcomes. Main endpoints were the analysis of progression-free survival. Progression-free survival was defined as the time from the first day of primary treatment until the date of progression, relapse or death for any cause, or last follow-up until December 31, 2024. Overall survival was defined as the time from the date of primary treatment to the date of death, or last follow-up.

### 2.2 Ethics considerations

This retrospective study was conducted in accordance with the ethical principles of the Declaration of Helsinki (World Medical Association, 2013) and was approved by the approval of the Ethics Committee of Beijing Chaoyang Hospital, Capital Medical University, Beijing, China. The requirement for informed consent was waived by the Ethics Committee of Beijing Chaoyang Hospital due to the retrospective nature of the study, and all data were anonymized to protect participant privacy.

### 2.3 Study participants

**Figure 1:**
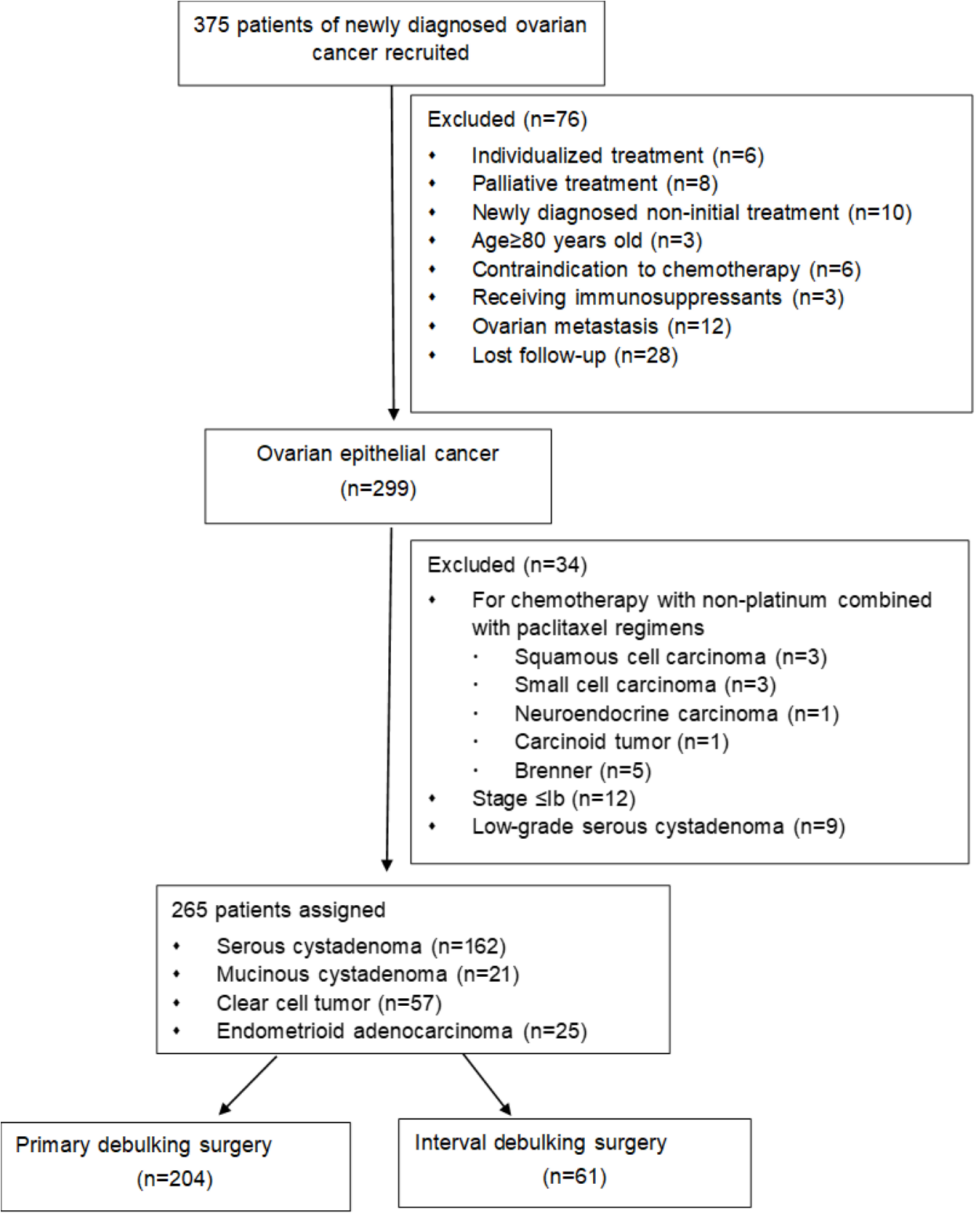
Flowchart of patients’ selection.

All patients diagnosed with ovarian cancer (FIGO stages IC to IV) (2) who underwent radical treatment, including standard cytoreductive surgery (either upfront primary debulking surgery or interval debulking surgery) and platinum-based adjuvant first-line chemotherapy were included. Patients in the primary debulking surgery group were required to have completed at least four cycles of postoperative platinum-based chemotherapy, whereas those in the intermediary cytoreductive surgery group must have received a minimum of two cycles of neoadjuvant chemotherapy. Participants who meet the inclusion criteria but encounter the following situations will be excluded from this study: (1) age <20 years or >80 years; (2) patients did not receive the above standardized treatment; (3) ovarian metastasis; (4) contraindications to chemotherapy, inability to complete standardized treatment or completion of fewer than 4 cycles of chemotherapy for other reasons; (5) patients received immunosuppressants (**Figure 1**).

### 2.4 Data collection

Baseline patient characteristics retrieved from medical records encompassed age at diagnosis, histologic subtype and differentiation, FIGO stage, Breast cancer susceptibility gene (BRCA)1/2 mutational status, type of surgery, residual disease status after surgery (residual disease 0 cm *vs*. 1cm/2cm), chemotherapy regimen, and serological level of carbohydrate antigen 125 at diagnosis. Biochemical indicators and complete blood cell count within one month before diagnosis was systematically collected as the baseline values for this study. If there were multiple results, the average was taken as the baseline value. Specialized personnel were responsible for managing and ensuring the accurate entry of follow-up data into the system.

The complete blood count parameters were examined via a hemogram autoanalyzer (Sysmex, Japan). The biochemical parameters were tested with an autoanalyzer (Siemens, USA).

This retrospective study was conducted in accordance with the ethical principles of the Declaration of Helsinki (World Medical Association, 2013) and was approved by the approval of the Ethics Committee of Beijing Chaoyang Hospital, Capital Medical University. The requirement for informed consent was waived by the Ethics Committee of Beijing Chaoyang Hospital due to the retrospective nature of the study, and all data were anonymized to protect participant privacy.

### 2.5 Statistical methods

Characteristics of the study population were summarized using descriptive statistical methods. Continuous variables are presented as the means ± SDs or medians and tertiles. Categorical data are reported as frequencies and percentages and were compared by means of the chi-square test or Fisher’s exact test, as appropriate. Differences according to the tertiles of platelets were compared by means of one-way analysis of variance for continuous data and chi-square tests for categorical variables.

We used a generalized additive model (GAM) to investigate the relationships between the baseline platelet count and progression-free survival with the recurrence rate. We used a logistic regression model to estimate the association between platelet count, morphology indices and progression-free survival. The results are presented as odds ratios (OR) with 95% confidence intervals (95% CI). Crude regression estimates, and estimates adjusted for covariates are presented. We selected these confounders on the basis of their association with the outcomes of interest or changes in effect estimates of more than 10%. After considering the clinical significance, we adjusted for the following covariates: age (years), type of surgery, residual disease at surgery (residual disease 0 cm *vs*. 1cm/2cm), cancer stage and pathology, indicators of routine blood tests and serum biochemical tests.

We then used a two-piecewise linear regression model to examine the threshold effect of platelets on progression-free survival with the recurrence rate. When Log-likelihood ratio test was less than 0.05, this implied a significant U-shaped relationship between the rate of recurrence and the basic platelet count. The turning point for the platelet was determined with “exploratory” analyses, which involve moving the trial turning point along the predefined interval and selecting the one that gave the maximum model likelihood. We used the bootstrap resampling method to calculate the 95% CI for the turning point, as described in previous analyses (17).

The two-sided alpha level was set at 0.05. All the statistical analyses were performed with SPSS software version 27.0 (IBM, Corp. in Armonk, NY, USA); R software, version 3.6.1 (R Project for Statistical Computing, Lucent, Mount Jasmine, New Jersey USA); and EmpowerStats (http://www.empowerstats.com, X and Y Solution, Inc., Boston, MA, USA) with IBM SPSS Statistics 20.0 (IBM Corporation, Somers, NY, USA).

## 3 Results

A total of 375 patients met the inclusion criteria during the study period. 110 cases met some exclusion criteria and were not included in the study. Ultimately, 265 patients with ovarian cancer were included in the analysis (**Figure 1**) and their characteristics are described in **Table 1**. The selection process for inclusion and exclusion patients is presented in **Figure 1**.

**Table 1:** Baseline characteristics of patients in ovarian cancer.

Characteristics	Total patients n=265
Age, n (%)	
<60	150 (56.90)
≥60	115 (43.10)
Diabetes mellitus, n (%)	
No	216 (82.13)
Yes	49 (17.87)
Hypertension, n (%)	
No	179 (67.55)
Yes	86 (32.45)
Stage, n (%)	
IC-II	82 (31.33)
III-IV	183 (68.67)
Histopathological type, n (%)	
Serous cystadenoma	162 (62.00)
Other types	103 (38.00)
Surgery, n (%)	
Intermediary cytoreductive surgery	61 (23.02)
Primary debulking surgery	204 (76.98)
Residual lesions, n (%)	
Yes	59 (22.30)
No	206 (77.67)
Venous thromboembolism, n (%)	
Yes	78 (29.43)
No	187 (70.57)
Recurrence, n (%)	
Yes	110 (41.51)
No	155 (58.49)

Among them, 204 patients underwent primary debulking surgery followed by a paclitaxel plus carboplatin chemotherapy regimen, and 61 patients received neoadjuvant chemotherapy followed by intermediary cytoreductive surgery and a postoperative paclitaxel plus carboplatin chemotherapy regimen. Patients in the primary debulking surgery group were required to receive 6 cycles of postoperative platinum-based chemotherapy, whereas those in the intermediary cytoreductive surgery group were required to have undergone a minimum of two neoadjuvant cycles and 4–6 cycles of postoperative platinum-based chemotherapy. The follow-up duration ranged from 4 to 134 months, with a median follow-up of 24.5 months. Among these cases, 110 patients experienced uncontrolled tumors, recurrence, or metastasis, while 155 patients remained progression-free. The median follow-up for the two groups were 14.1 months and 47 months, respectively. Progression-free survival rate at 3 years was 57.73% (95% CI 51.27-65.0%) and at 5 years was 50.29% (95% CI 43.42-58.26%).

### 3.1 Patients’ baseline characteristics

Data from 265 ovarian cancer patients were analyzed. The baseline characteristics, routine blood test results and biochemical indicators of the 265 patients diagnosed with ovarian cancer are presented in **Table 1**. The median age was 57 years (range 37–76 years). There were 49 (16.33%) patients with diabetes mellitus and 86 (32.45%) patients with hypertension. A total of 162 (61.13%) patients had high grade serous cystadenomas. The majority of the patients had advanced-stage disease (stage III-IV: 193, 72.83%) and high histological grade (grade 3: 77.67%) (**Table 1**).

### 3.2 Identification of nonlinear relationships

Examination of the association between baseline platelet count and ovarian cancer outcomes in univariate analyses indicated that the platelet count is significantly associated with the risk of ovarian cancer recurrence (OR = 1.02, 95%CI 1.01-1.04, *P* = 0.0001). The recurrence risk differs statistically significant across distinct platelet count ranges by interaction analysis (*P* = 0.0072), a U-shaped association between baseline platelet count and recurrence risk was identified (*P* <  0.05) (**Table 2**).

**Table 2:** Stratified analysis of platelet count and the risk of recurrence of ovarian cancer.

Exposure	OR of recurrence (95%CI)	*P*-value	*P*-interaction
Platelet count	1.02(1.01-1.04)	0.0001	
Platelet count Tertile			0.0072
Low	reference	1.0000	
Middle	0.88(0.57-1.53)	0.6300	
High	2.15(1.35-3.43)	0.0013	

**Figure 2:**
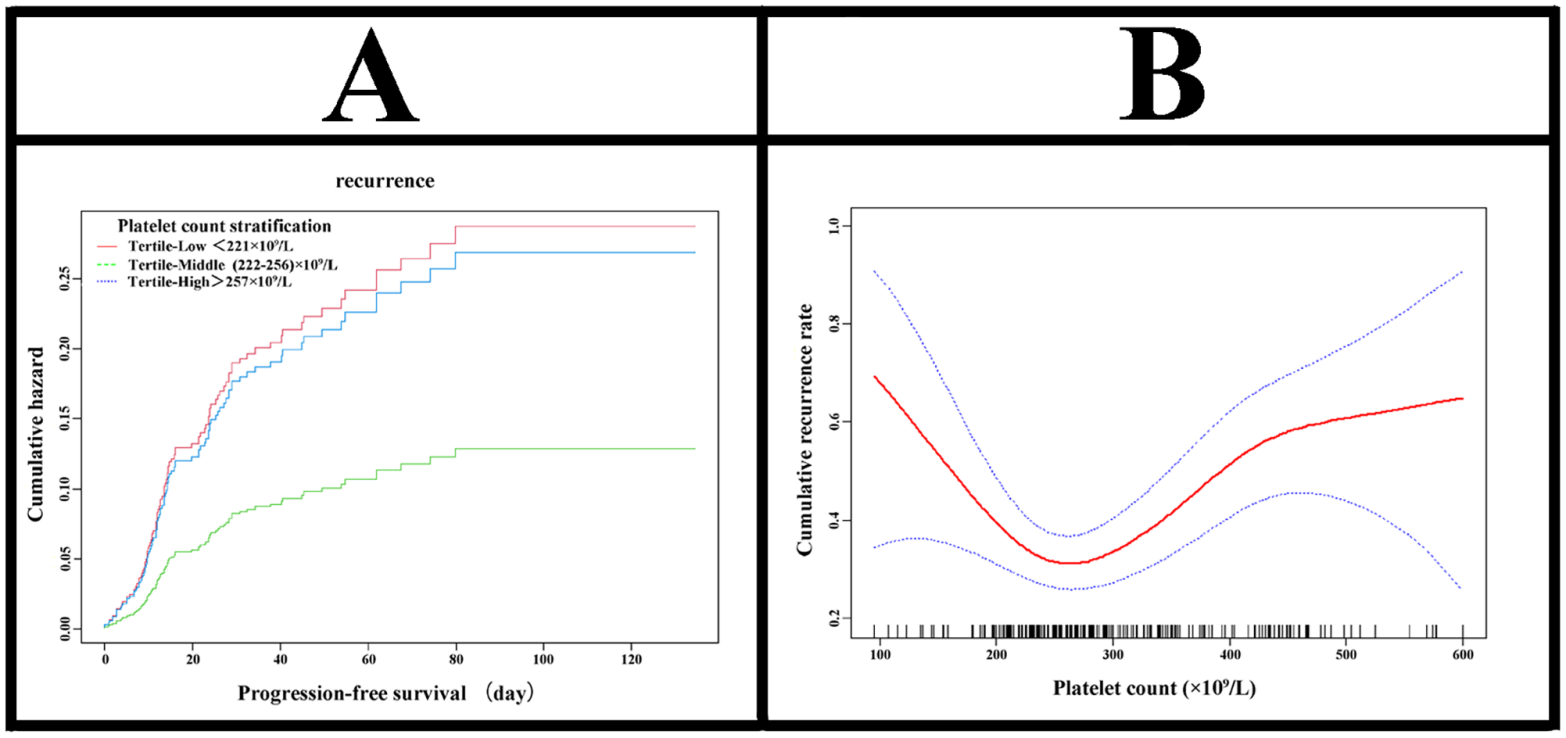
Association between baseline platelet count and prognosis in ovarian cancer. **(A)** displays a cumulative hazard plot showing progressionfree survival by platelet count tertiles, with middle platelet counts (222-256×10^9^/L) linked to lowest recurrence hazard. **(B)** presents a line chart depicting cumulative recurrence rate versus platelet count, indicating a U-shaped relationship with lowest recurrence at midrange platelet counts and confidence intervals in blue.

The analysis of the smooth curve and threshold effect revealed that the baseline platelet count exhibited a U-shaped relationship with posttreatment recurrence risk (**Figure 2B**). The turning point of the platelet count was 236×10^9^/L (95% CI: 222-256×10^9^/L) (**Supplementary Table S1**). The recurrence rate curves for platelet counts ≤ 221×10^9^/L, 222-256×10^9^/L and > 256×10^9^/L are shown in **Figure 2A**.

### 3.3 Association between baseline platelet count and the risk of ovarian cancer recurrence

A U-shaped association between baseline platelet count and the risk of ovarian cancer recurrence was observed in Multinomial logistic regression after adjustment for potential confounders and covariates (**Table 3**). The association between platelet and platelet morphology indices with the recurrence of ovarian cancer was analyzed by multiple regression models.

**Table 3:** Multinomial logistic regression of platelet count and the risk of recurrence of ovarian cancer.

Variables	Non-adjusted	Adjust I	Adjust II
**Platelet count**	1.00 (1.00, 1.00) 0.0042	1.00 (1.00, 1.01) 0.0034	1.00 (1.00, 1.01) 0.0106
Platelet Tertile			
**Low**	1.0	1.0	1.0
**Middle**	0.88 (0.52, 1.49) 0.6268	0.71 (0.37, 1.37) 0.3094	0.58 (0.23, 1.50) 0.2619
**High**	2.15 (1.35, 3.43) 0.0013	2.02 (1.07, 3.82) 0.0305	2.65 (1.01, 6.94) 0.0475

Adjust I adjust for: pathologic type; age; diabetes mellitus (0, 1); neoadjuvant chemotherapy (0, 1); venous thromboembolism (0, 1); albumin; high-density lipoprotein; mean platelet volume; platelet distribution width; platelet large cell ratio; prealbumin; alkaline phosphatase; lactate dehydrogenase; gamma-glutamyl transferase; uric acid; indirect bilirubin.

Adjust II adjust for: pathologic type; age (smooth); diabetes mellitus (0, 1); neoadjuvant chemotherapy (0, 1); venous thromboembolism (0, 1); albumin (smooth); high-density lipoprotein (smooth); mean platelet volume (smooth); platelet distribution width (smooth); platelet large cell ratio (smooth); prealbumin (smooth); alkaline phosphatase (smooth); lactate dehydrogenase (smooth); gamma-glutamyl transferase (smooth); uric acid (smooth); indirect bilirubin (smooth).

### 3.4 The association between platelet morphological indicators and prognosis of ovarian cancer

**Figure 3:**
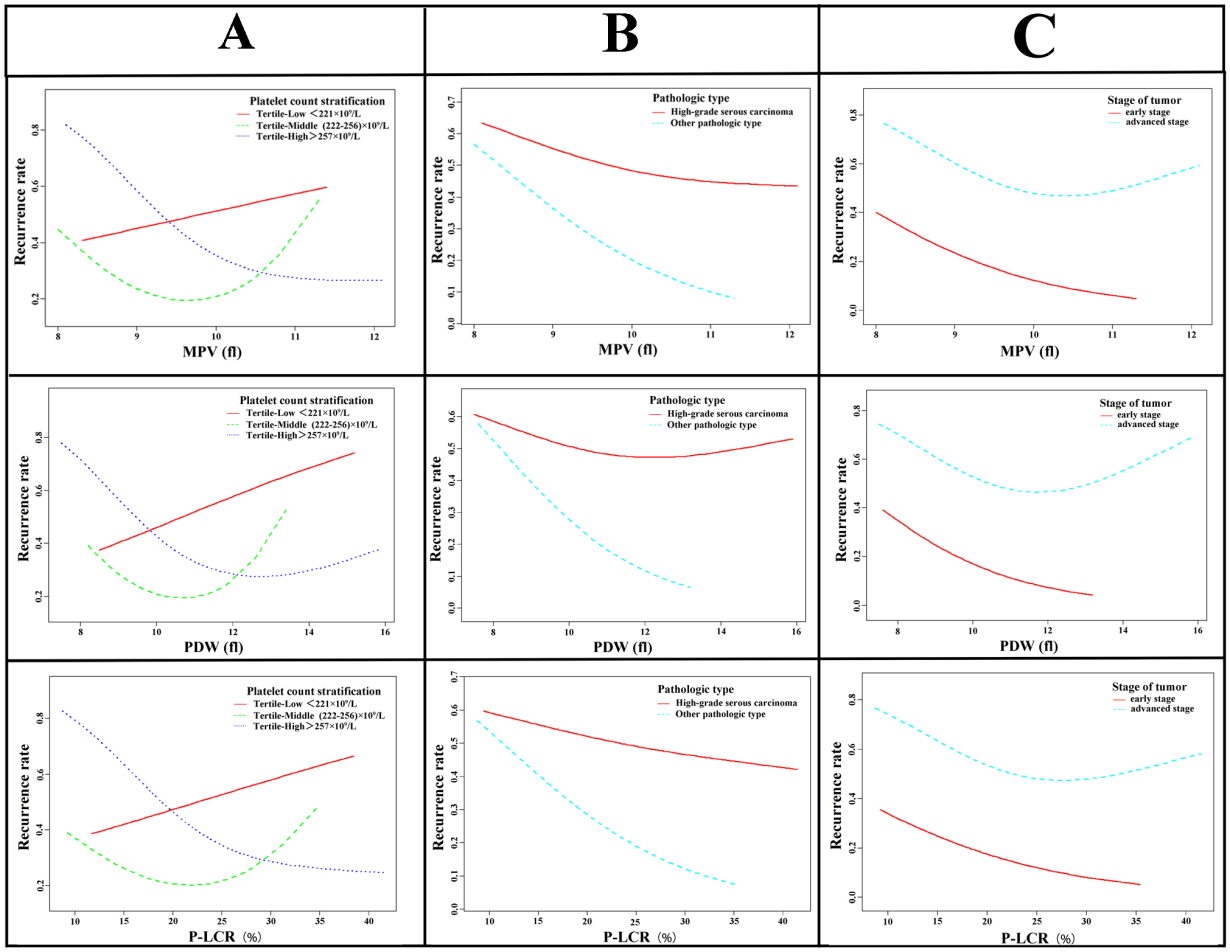
Relationship between baseline platelet morphological indices and prognosis of ovarian cancer. Grid of nine line charts divided into columns labeled **(A–C)**. Each column examines platelet count relationships, with rows representing PDW, MPV, and PLCR values. The leftmost column compares remission and relapse recurrence rates; the middle compares high-grade serous carcinoma with other pathological types; the right compares early versus advanced tumor stages. Red and blue dashed lines represent subgroup trends in each chart.

A stratified analysis was conducted based on baseline platelet count, the relationship between baseline platelet morphological indices and prognosis was analyzed. Platelet morphological indicators were associated with prognosis and exerted a protective effect as platelet count was in the third tertile. Histological subtype stratification revealed that morphological indicators were not associated with the risk of recurrence in high-grade serous carcinoma. However, these values were associated with the recurrence risk in other pathological types, with higher values corresponding to lower recurrence risk (**Figure 3**, **Supplementary Table S2**, **S3**). Stratified clinical staging was not associated with platelet morphological indices.

Non-parametric Mann-Whitney U test showed that there was no significant difference in baseline platelet count between the disease recurrence group and the remission group in patients with high-grade serous carcinoma and other pathological types (*P* = 0.153 and *P* = 0.359, respectively). In patients with high-grade serous carcinoma, there was no statistically significant difference in baseline PDW, MPV and PLCR between the recurrence group and the remission group (*P* > 0.050, respectively). However, in patients with other pathological types, there were significant differences in the above three morphological indicators between the disease remission group and the recurrence group (PDW, *P* = 0.0024; MPV, *P* = 0.0053; PLCR, *P* = 0.0056).

The association between platelet morphology indicators and prognosis is consistent with the findings from univariate analysis. It illustrates that the relationship between platelet morphology indicators and clinical outcomes according to baseline platelet levels (**Figure 3**). The baseline platelet count range associated with the lowest risk of recurrence is 222-256×10^9^/L. In patients with baseline platelet count below 221×10^9^/L, the risk of recurrence increases as morphology indicators rise; in contrast, among those with platelet count exceeding 256×10^9^/L, higher morphology indicator values are associated with a reduced risk of recurrence, a pattern consistent with a U-shaped relationship.

The relationship between three morphological indicators and recurrence in patients with high-grade serous carcinoma and other pathological types is illustrated in **Figure 3B**. As the values of MPV, PDW and PLCR gradually increase, the recurrence rate of patients with high-grade serous carcinoma does not change significantly. While for other pathological types, the recurrence rate decreases rapidly with increasing values of MPV, PDW and PLCR (MPV, *P* = 0.0706; PDW, *P* = 0.0704; PLCR, *P* = 0.0765).

The relationship between three platelet morphological indicators and recurrence in patients with advanced and early stage ovarian cancer is illustrated in **Figure 3C**. The curves of morphological indicators and recurrence rate in early and advanced stage patients are similar in shape, but the recurrence rate in advanced stage cancer is significantly higher.

### 3.5 The relation of platelet count and morphological parameters of platelets, and the relation and prognosis of ovarian cancer

**Figure 4:**
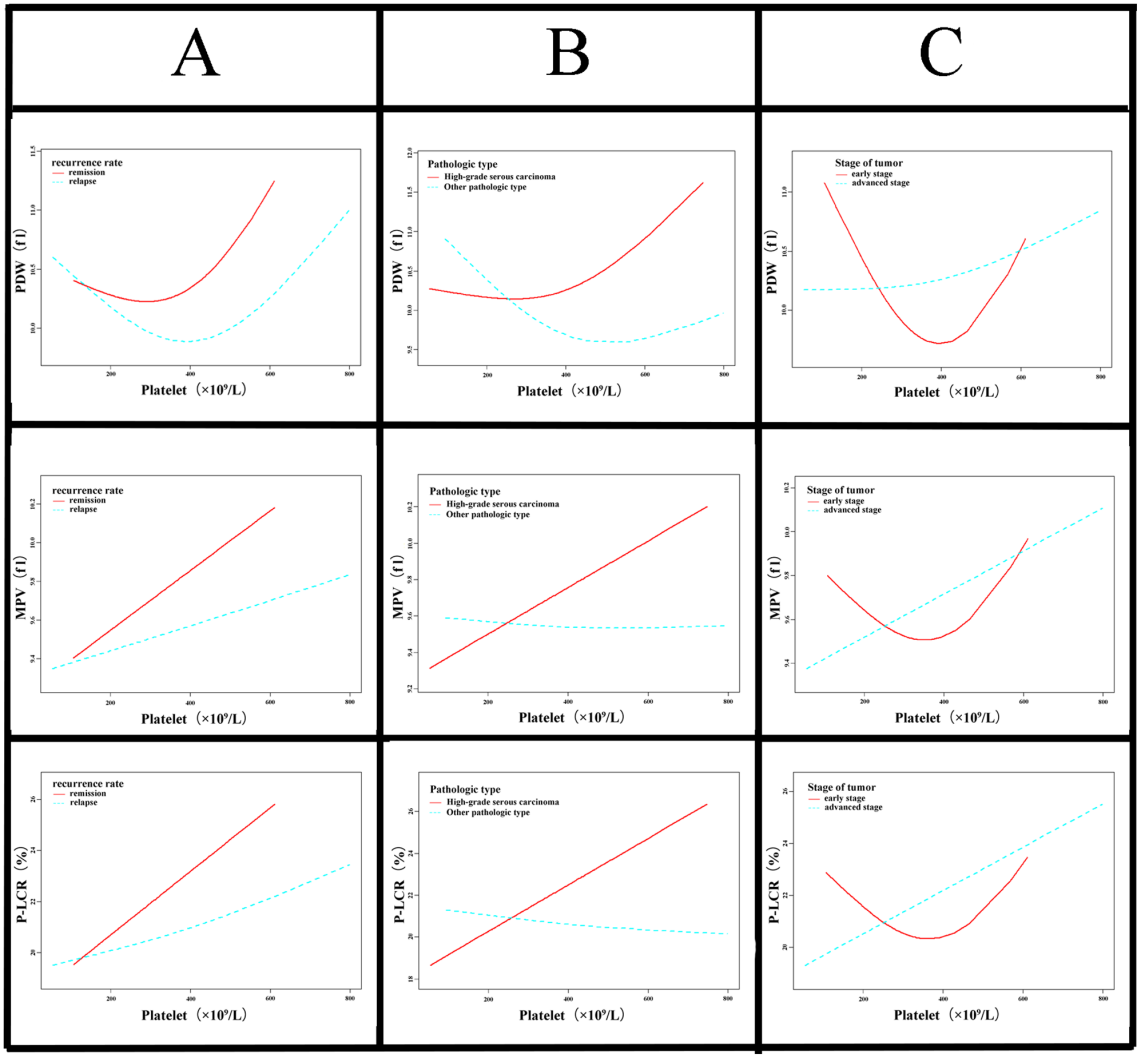
Association between baseline platelet morphological indices and platelet count in ovarian cancer according to histopathological subtype and clinical stage. **(A)** Trend of platelet morphology indices and platelet count is associated with clinical prognosis. The smooth curve indicates that the baseline morphological indices increase as the baseline platelet count rises. Compared with patients with tumor recurrence, the increase in platelet morphological indicators was greater in patients with tumor remission (PDW, *P* = 0.048; MPV, *P* = 0.0212; PLCR, *P* = 0.0318). **(B)** It presents the stratified analysis of histopathological subtypes. The morphological indicators progressively increase with rising platelet levels in high-grade serous carcinoma (red line). whereas in other pathological types (blue line), these indicators exhibit a slight decline as platelet counts increase. Patients with high-grade serous carcinoma exhibit a distinct trend in platelet morphology indices to other pathological subtypes, although the statistical difference is not significant (PDW, *P* = 0.3194; MPV, *P* = 0.328; PLCR, *P* = 0.388). **(C)** The morphological indicators show a continuous upward trend with increasing platelet counts in advanced stage of ovarian cancer. The research indicates that there is no significant statistical difference in the trends of the morphological indices as platelet count increases between advanced stage group and early stage group (PDW, *P* = 0.4416; MPV, *P* = 0.5557; PLCR, *P* = 0.4811).

Whether the pathological type and clinical stage affect the pattern of dynamic changes in platelet volume with platelet count, we analyzed the dynamic changes of platelet count and their morphological indicators separately in high-grade serous carcinoma *vs* other pathological types of ovarian cancer, and in early stage ovarian cancer *vs* advanced stage ovarian cancer. As shown in **Figure 4**, among patients with high-grade serous carcinoma, there was no significant difference in three platelet morphological indicators between relapsed and remission patients as platelet levels increased. However, for patients with other pathological types, relapsed patients showed significantly lower morphological indicators than remission patients as platelet levels rose (**Supplementary Table S3**). The morphological indicators dynamic changes between relapsed and remission are no significantly different, not only early stage cancer, but also advanced stage cancer.

## 4 Discussion

Most studies have reported a linear correlation between platelet count and the prognosis of tumors. However, a few studies have reached different conclusions. In a Danish registry-based cohort study, it was found that low and high platelet count were significantly associated with decreased overall survival in non-small-cell lung cancer patients (18). It is rarely reported that the U-shaped curve correlation between pre-treatment platelet count and the prognosis of ovarian cancer. This study confirmed through multi-dimensional analysis that the baseline platelet count has a significant U-shaped relationship with the prognosis of ovarian cancer. Stratification analysis showed that the risk of recurrence varies in different platelet stratified, with a smooth curve revealing a U-shaped curve. Threshold effect analysis identified the baseline platelet range with the lowest recurrence risk. The predictive value of baseline platelet count for ovarian cancer recurrence risk was confirmed after excluding the interference of confounding factors and the effects of covariates by multinomial logistic regression. The low-risk platelet range is narrow. Survival analysis showed that baseline platelet count in this small range has a significantly lower recurrence rate than the ranges on either side. When analyzing the relationship between platelet morphological indicators and ovarian cancer prognosis, we observed that the morphological indicators corresponding to the three segments of the U-shaped curve-defined by baseline platelet count and recurrence risk are significantly associated with patient outcomes. In the left limb of the U-shaped curve (low platelet count range), an increase in morphological indicators is associated with a higher risk of recurrence.

The cytoplasm of platelets is rich in functional proteins, organelles and granules. Various receptors are densely distributed on the platelet membrane, and upon activation, they will trigger a complex intracellular signal cascade reaction (19). Through these mechanisms, platelets are integral to critical physiological processes such as thrombosis formation, wound healing and immune surveillance. The low platelet count in cancer patients may be due to the consumption of platelets by the tumor. This can lead to poor tolerance to anti-tumor treatment, an increased risk of bleeding, and a poor prognosis. Previous studies have shown that platelets help tumors evade immune surveillance by forming “platelet-tumor cell microthrombi” with circulating tumor cells. The soluble mediators released by platelets, such as adenosine diphosphate, thromboxane A2, and prostaglandin E2, exert their effects through G protein-coupled receptors leading to more platelet activation and driving the metastasis signaling pathways of cancer cells (20). There is evidence suggesting that platelets and the released TGF-β can protect tumor cells from chemotherapy drugs. Therefore, as the platelet count on the right side of the U-shaped curve increases, the risk of tumor recurrence in patients increases. It is recommended that the physiological functions of platelets are maintained, while their pathological promotion of tumor metastasis has not been significantly activated in the middle interval of the U-shaped curve. This reflects that the patient has a relatively small tumor burden and a stable overall condition.

In contrast, during the middle and right segments, further increases in morphological indicators are linked to a rapid decline in recurrence risk. This bidirectional pattern of morphological indicators in relation to recurrence risk exhibits a distinct U-shaped trend, providing further confirmation of the U-shaped association between platelet parameters and clinical prognosis.

The second finding of this study indicates that the recurrence risk of ovarian cancer is closely related to platelet morphological indicators. The changing trends of these morphological parameters are associated with baseline platelet levels. Among the patients with ovarian cancer who did not experience recurrence during the follow-up period, the baseline PDW, MPV and PLCR, three platelet morphological indicators, were significantly higher than those of the patients with recurrence. Additionally, platelet morphological indicators are associated with the recurrence risk of other pathological types such as non-high-grade serous papillary carcinoma. Previous studies on the relationship between platelets and the prognosis of malignant tumors have mostly focused on inflammatory-related indicators, mainly examining platelet counts or single morphological parameters. There are no systematic integration and in-depth analysis of the overall dynamic changes in platelets (11, 17, 21–23).

When the platelet count of patients with ovarian cancer shows a U-shaped curve relationship with the risk of recurrence, the dynamic changes in platelet morphological indicators are closely related to the baseline platelet level. In different platelet count intervals, the relationship between the trend of platelet morphological changes and the risk of recurrence varies: within the lower range of platelet counts, as platelet morphological indicators (such as PDW and MPV) increase, the risk of recurrence also increases; however, when the platelet count moves out of the low range, further increases in morphological indicators are associated with a decreased risk of recurrence. The changes in platelet count and morphological parameters not only reflect the tumor burden, but also indicate the status of the host’s bone marrow and systemic response. When the platelet count is at a low level, it may be due to factors such as tumor disease progression consuming platelets, poor nutritional status of the patient, and suppression of bone marrow hematopoietic function. Although compensatory “platelet morphological activation” occurs; the poor prognosis is difficult to change. Under the condition that the platelet count remains at a high level, its morphological activation may reflect the physical superior ability to cope with the tumor and the reserve function of the bone marrow. It is speculated that to a certain extent, it buffers the malignant progression of the tumor, and therefore is related to a relatively low recurrence rate. To some extent, a low recurrence rate is related to the body’s compensatory ability buffers the malignant progression of the tumor. The above findings still require further basic research and clinical observation for verification.

This study advances understanding and promotes the clinical translation of platelet biology in oncology. From a clinical perspective, the platelet characteristics of patients with poor prognosis will guide individualized treatment strategies, poor prognosis patients with low platelet counts may receive downgraded chemotherapy regimens to reduce toxicity, while patients with poor prognosis and high platelet count may benefit from intensified anti-angiogenic therapy, thrombocythemia and so on. Due to their central involvement in tumor metastasis, platelets have become prominent targets for therapeutic intervention. The current treatment methods mainly involve anti-tumor treatment through anti-platelet therapy. Highlighting the role in both physiological and pathological contexts will help us further explore the clinical therapeutic potential of platelets. With the continuous development of platelet biology, new treatment strategies focus on improving platelet regulation to enhance the clinical efficacy of tumor treatment and prevent complications related to platelet dysfunction.

### 4.1 Strengths and limitation

The present study is limited by its retrospective single-center design and the absence of genetic mutation data integration, highlighting the necessity for validation through prospective cohort studies. Although the statistical modeling is robust, the lack of external validation should be more explicitly acknowledged. Future initiatives will prioritize the development of a dynamic monitoring system encompassing both platelet count and function to facilitate real-time prediction of recurrence risk. Moreover, this study revealed the association between the recurrence risk of ovarian cancer patients and platelet count as well as platelet morphological characteristics, and the relationship between the dynamic changes of morphological indicators with platelet count and recurrence risk. However, the study did not involve the detection of related cytokines, thus it was unable to deeply explore the underlying biological mechanisms. Future prospective studies are needed to further clarify the molecular mechanisms behind platelet count and platelet morphological changes, and to identify potential intervention targets, providing a scientific basis for the early diagnosis and development of new treatment strategies for ovarian cancer.

## 5 Conclusion

This study’s findings reveal a significant nonlinear association between the recurrence risk of ovarian cancer patients and platelet count as well as platelet morphological characteristics. It showed a pattern consistent with a U-shaped relationship. The baseline platelet count range of 222-256×10^9^/L was associated with the lowest risk of recurrence. Outside this range, both the low-level and high-level platelet ranges were associated with a high risk of tumor recurrence. Platelet is a meaningful prognostic indicator and therapeutic target in clinical practice. Future studies should explore the intricate mechanisms that connect baseline platelet characteristics and ovarian cancer outcomes. Precise regulation of platelet will bring prospects to cancer treatment.

## Data Availability

The original contributions presented in the study are included in the article/**Supplementary Material**. Further inquiries can be directed to the corresponding authors.
