# Supplementary material for: U-shaped prognostic signature: baseline platelet count and morphological parameters predict ovarian cancer outcomes in a 265-patient cohort
Source: Front Oncol. 2026 Feb 13;16:1765566. doi: 10.3389/fonc.2026.1765566 (PMC12945817; doi:10.3389/fonc.2026.1765566)
Supplement: Supplementary file 1 [file Table1.docx]

**S-Table 1. Threshold effect analysis of the risk of platelet count and cancer recurrence**

| **Platelet** | **Unadjusted** | | **Adjusted*** | |
| --- | --- | --- | --- | --- |
| **Models** | OR (95%CI) | *P* value | OR (95%CI) | *P* value |
| **Model I** | | | | |
| **One line effect** | 1.0 (1.0, 1.0) | 0.013 | 1.0 (1.0, 1.0) | 0.076 |
| **Model II** | | | | |
| **Turning point (K)** | 235×10^9^/L |  | 236×10^9^/L |  |
| **Platelet < K** | 1.0 (1.0, 1.0) | 0.008 | 1.0 (1.0, 1.0) | 0.0025 |
| **Platelet ≥ K** | 1.0 (1.0, 1.0) | <0.001 | 1.0 (1.0, 1.0) | 0.0017 |
| ***P* value for LRT test*** | 0.0030 |  | 0.0037 |  |
| **95% CI of K** | 221, 255 |  | 225, 256 |  |

Abbreviations: CI: confidence interval; OR: odds ratio; LRT: logarithm likelihood ratio test.

Data were presented as OR (95% CI) and *P* value. Model I: linear analysis; Model II: non-linear analysis. *: *P*< 0.05 indicates that model II is significantly different from Model I.

*Adjusted for age (years), stage, type of pathology, diabetes melitus, blood pressure.

**S-table 2 The trend of platelet morphological changes with variations in platelet count ≥257×10^9^/L**

| **Platelet count**  **（≥****257×10^9^/L）** | **Free** | **Recurrent** | **Standardize diff** | ***P-* value** |
| --- | --- | --- | --- | --- |
| **PDW** | 10.3 ± 1.4 | 9.8 ± 1.6 | 0.3 (0.0, 0.7) | 0.038 |
| **MPV** | 9.8 ± 0.7 | 9.5 ± 0.8 | 0.4 (0.1, 0.7) | 0.013 |
| **PLCR** | 22.7 ± 5.9 | 20.2 ± 6.4 | 0.4 (0.1, 0.7) | 0.017 |

Abbreviations: MPV: mean platelet volume, PDW: platelet distribution width, PLCR: large platelet ratio.

In the subgroup with platelet count ≥ 257×10^9^/L (166 case including 75 relapsed cases), the morphological indicators of relapsed patients were all significantly lower than those of patients of remission.

**S-table 3 Comparison of** **platelet morphological Indicators in high-grade serous carcinoma and other pathological types between recurrence and remission**

| **Morphological Indicators** | **Pathological type** | **Recurrence** | **Mean** | **Sd** | **P** |
| --- | --- | --- | --- | --- | --- |
| **MPV** | High-grade serous carcinoma | 0 | 9.8383 | 0.7671 | 0.140*^1^ |
|  | High-grade serous carcinoma | 1 | 9.5774 | 0.8192 | 0.658^#1^ |
|  | Other pathological types | 0 | 9.7515 | 0.6838 | 0.0037*^2^ |
|  | Other pathological types | 1 | 9.0857 | 0.6769 | **0.043^#2^** |
| **PDW** | High-grade serous carcinoma | 0 | 10.4277 | 1.4673 | 0.2004*^1^ |
|  | High-grade serous carcinoma | 1 | 10.0302 | 1.6000 | **0.576^#1^** |
|  | Other pathological types | 0 | 10.2424 | 1.3349 | 0.0080*^1^ |
|  | Other pathological types | 1 | 9.1143 | 1.1183 | **0.048^#2^** |
| **PLCR** | High-grade serous carcinoma | 0 | 23.0468 | 6.1314 | 0.1257*^1^ |
|  | High-grade serous carcinoma | 1 | 21.0868 | 6.5071 | **0.6453^#1^** |
|  | Other pathological types | 0 | 22.3303 | 5.6160 | 0.0043*^1^ |
|  | Other pathological types | 1 | 17.0714 | 5.1443 | **0.0365^#2^** |

*1 Comparison of three morphological indicators for recurrence and remission in patients with high-grade serous carcinoma (**platelet count**≥257×10^9^/L)

*2 Comparison of three morphological indicators for recurrence and remission in patients with other pathological types (**platelet count**≥257×10^9^/ L）

#1 Comparison of three morphological indicators between high-grade serous carcinoma and other pathological types in disease remission (**platelet count**≥257×10^9^/L)

#2 Comparison of three morphological indicators between high-grade serous carcinoma and other pathological types in recurrence (**platelet count**≥257×10^9^/L)
